# [Retracted] MicroRNA‑744 suppresses cell proliferation and invasion of papillary thyroid cancer by directly targeting NOB1

**DOI:** 10.3892/mmr.2023.13066

**Published:** 2023-08-07

**Authors:** Haixia Liu, Jing Guo, Hongyan Chai, Xiangfeng Meng

Mol Med Rep 19: 1903–1910, 2019; DOI: 10.3892/mmr.2019.9826

Following the publication of this paper, it was drawn to the Editor's attention by a concerned reader that certain of the cell migration and invasion assay data shown in Fig. 5C were strikingly similar to data appearing in different form in other articles by different authors at different research institutes. Owing to the fact that the contentious data in the above article were already under consideration for publication, or had already been published, prior to its submission to *Molecular Medicine Reports*, the Editor has decided that this paper should be retracted from the Journal. The authors were asked for an explanation to account for these concerns, but the Editorial Office did not receive a reply. The Editor apologizes to the readership for any inconvenience caused.

